# Enhancement of Paclitaxel's Anticancer Efficacy in Thyroid Cancer by Isodeoxyelephantopin Through Modulation of Oxidative Stress

**DOI:** 10.1002/fsn3.71266

**Published:** 2025-12-12

**Authors:** Wei Cong, Jingfu Sun, Zhanyu Hao, Maosong Gong

**Affiliations:** ^1^ Department of Thyroid Surgery The Second Hospital of Shandong University Jinan Shandong China

**Keywords:** chemosensitizing effect, isodeoxyelephantopin, oxidative stress, p62‐Keap1‐Nrf2, paclitaxel

## Abstract

This study demonstrates that IDET enhances the efficacy of paclitaxel (taxol) against thyroid cancer by inducing reactive oxygen species (ROS)–mediated apoptosis. IDET synergizes with taxol to inhibit proliferation, migration, and invasion of thyroid cancer cells while sparing normal thyroid cells from cytotoxic effects. Mechanistically, the combination treatment downregulates p62 and upregulates Keap1, leading to suppression of Nrf2 signaling and antioxidant gene expression. This disruption of redox homeostasis leads to elevated ROS levels, which trigger apoptosis. In vivo, IDET significantly enhances the antitumor effects in a thyroid cancer xenograft mouse model, resulting in increased oxidative stress and apoptosis through inhibition of the p62‐Keap1‐Nrf2 pathway. Importantly, IDET exhibits minimal toxicity to major organs and does not adversely affect hematological parameters, indicating a favorable safety profile. These findings suggest that targeting the p62‐Keap1‐Nrf2 axis to disrupt redox balance represents an effective strategy to overcome chemoresistance. IDET emerges as a promising chemosensitizer for improving therapeutic outcomes in thyroid cancer treatment.

## Graphical abstract

Isodeoxyelephantopin (IDET) enhances paclitaxel sensitivity in thyroid cancer by promoting ROS‐mediated apoptosis through inhibition of the p62‐Keap1‐Nrf2 antioxidant pathway. The combination treatment suppresses tumor growth and metastasis with minimal toxicity, suggesting IDET as a safe and effective chemosensitizer to improve Taxol‐based therapy.

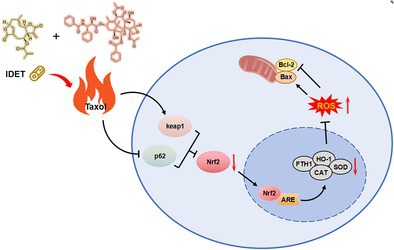

## 1 Introduction

Thyroid cancer is a prevalent malignancy in head and neck surgery, and its incidence has risen steadily worldwide over the last 30 years (Han et al. 2024; Liu et al. 2023). Surgery is the most recommended treatment for thyroid cancer, followed by adjuvant radiotherapy and thyroid hormone replacement therapy (Filetti et al. 2019). Thyroid cancers are classified into different types on the basis of their origin and biological characteristics, and papillary thyroid carcinoma (PTC) is the most prevalent type, accounting for 85%–90% of cases. PTC is generally controlled through standardized treatment strategies, such as surgery combined with postoperative radioactive ^131^I therapy. By contrast, anaplastic thyroid cancer is highly aggressive and has a poor prognosis, and effective treatments remain elusive (Califano et al. 2023). Paclitaxel (taxol) possesses antitumor activity against various types of cancer, including breast, ovarian, lung, esophageal, and head and neck cancer (Yang et al. 2020). Taxol disrupts normal microtubule dynamics required for cell division and critical interphase processes by promoting tubulin polymerization, leading to tumor cell death. However, resistance to chemotherapeutic agents, such as taxol, poses a major challenge to the treatment of highly malignant thyroid cancers. Therefore, the development of novel synergistic drugs is crucial to reducing the side effects of taxol, optimizing its pharmacological properties, and enhancing its antitumor efficacy.

Reactive oxygen species (ROS), which are the byproducts of oxygen metabolism, play a significant role in cancer initiation, progression, and metastasis. Compared with normal cells, tumor cells consistently maintain a higher redox state. Throughout tumor development, these cells often exhibit multiple genetic mutations and elevated oxidative stress, which when excessive, can disrupt redox homeostasis (Jelic et al. 2021). Modern pharmacological research has shown that the intrinsic pathway of apoptosis is mediated by the increased permeability of the mitochondrial outer membrane, and many forms of apoptosis are associated with the loss of mitochondrial membrane potential (Rehfeldt et al. 2021). The accumulation of ROS within cells can lead to mitochondrial depolarization, which subsequently activates the intrinsic apoptotic pathway and drives tumor cell apoptosis (Srinivas et al. 2019). Current studies have confirmed a close association between ROS and thyroid cancer development (Wang et al. 2019). Consequently, anticancer therapies can effectively eliminate tumors by promoting ROS generation within cells.

The Keap1‐Nrf2 pathway protects the body from oxidative stress by activating genes that encode antioxidants, detoxifying enzymes, metabolic enzymes, and transporters, and enhancing cellular defenses against oxidative and xenobiotic stress (Sykiotis 2021). This pathway is often dysregulated in human cancers, resulting in the abnormal activation of Nrf2 (Krajka‐Kuźniak et al. 2017). Knocking out Nrf2 leads to a marked decrease in tumor cell proliferation (Kitamura et al. 2017). p62, which is a key regulatory factor in the Keap1‐Nrf2 pathway, prevents Keap1 from binding to Nrf2, thereby inhibiting the ubiquitination and degradation of Nrf2, which stabilizes Nrf2 and enhances the expression of its downstream target genes (Ryoo et al. 2018; Yamada et al. 2019). This pathway can lead to abnormal Nrf2 accumulation in tumor cells, which provides protection against apoptosis, promotes tumor growth, and induces chemoresistance in cancer cells (Ning and Wang 2019). Hence, Nrf2 has emerged as a potential therapeutic target in cancer treatment, and natural compounds, such as quercetin and luteolin, induce apoptosis in cancer cells by inhibiting Nrf2 (Mostafavi‐Pour et al. 2017; Wang et al. 2017). Given that the abnormal activation of Nrf2 is associated with thyroid cancer progression and chemoresistance (Li et al. 2021), targeting the Keap1‐Nrf2 pathway is considered a promising anticancer strategy.

The extraction of small‐molecule compounds from natural plants for disease treatment is a crucial approach in the development of anticancer drugs because of these compounds' excellent safety profiles. Deoxyelephantopin (DET) and isodeoxyelephantopin (IDET) are sesquiterpene lactones derived from the whole herb of *Elephantopus scaber* , which is a member of the Asteraceae family known for its hepatoprotective, antibacterial, and antiviral properties. DET possesses anticancer properties against various types of cancer, inhibiting tumor cell proliferation and migration, inducing apoptosis, and enhancing the sensitivity of tumor cells to chemotherapeutic agents. For instance, DET induces apoptosis in hepatocellular carcinoma cells through oxidative stress, NF‐κB inhibition, and mitochondrial dysfunction (Xu et al. 2017). Additionally, DET exhibits activity against triple‐negative breast cancer cells by inhibiting extracellular signaling and protein functions through ROS‐mediated mechanisms (Ren et al. 2017; Zhang et al. 2018). Furthermore, it inhibits the lung metastasis of TS/A mouse mammary carcinoma cells (Chen et al. 2017). Several studies have underscored the safety of DET, which induces toxicity that is specific to cancer cells (Cheikh et al. 2022). However, the use of IDET in the treatment of thyroid cancer has not been reported, and whether IDET can exert anticancer effects by synergizing with taxol, as well as and the underlying mechanisms, remain unclear.

Thus, this study explores how IDET enhances the anti‐thyroid cancer effects of taxol. We demonstrated that ROS play a crucial role in the anti‐thyroid cancer effects of IDET, and our findings revealed that IDET promotes the anti‐thyroid cancer activity of taxol through the modulation of the p62‐Keap1‐Nrf2 pathway with ROS. Furthermore, the in vivo effectiveness of IDET in promoting the anti‐thyroid cancer activity of taxol was confirmed, and potential organ toxicity was evaluated. These findings provide scientific support for the clinical application of IDET and offer novel targets and strategies for the development of therapeutic agents for thyroid cancer.

## 2 Methods and Methods

### 2.1 Cell Culture

Human thyroid normal Nthy‐ori 3‐1 cells, and thyroid cancer cell lines TCP‐1 and BCPAP were obtained from the American Type Culture Collection (USA). Nthy‐ori 3‐1 and TCP‐1 cells were cultured in RPMI‐1640, while BCPAP cells were cultured in DMEM. All cell lines were maintained in media supplemented with 10% fetal bovine serum (FBS), 1% penicillin–streptomycin, and incubated at 37°C in a humidified atmosphere containing 5% CO_2_. All cell lines were authenticated using short tandem repeat profiling and were routinely screened for mycoplasma contamination. Taxol‐resistant TCP‐1 and BCPAP cells (TCP‐1/taxol and BCPAP/taxol) were developed by gradually increasing the concentration of taxol (from 0.1 to 500 nM) over a period of 4 months, resulting in the successful establishment of drug‐resistant cell lines.

### 2.2 Cell Viability Assay

Cells (3000/well) were seeded in 96‐well plates and treated with either vehicle control (DMSO) or various concentrations of IDET or taxol. After incubation for 24 h, 10 μL of CCK‐8 reagent was added for an additional 2 h. The absorbance at 450 nm was measured using a microplate reader to determine the optical densities. The half‐maximal inhibitory concentration for each group was then calculated.

### 2.3 Colony Formation, Wound Healing, and Transwell Invasion Assays

For the colony formation assay, cells (1000/well) were seeded into six‐well plates. The medium was refreshed every 3 days, and cells were cultured for 10–12 days. After incubation, the medium was discarded, and the cells were fixed with formaldehyde, stained with 0.5% crystal violet for 10 min, and then photographed. The colonies were then counted.

For the wound healing assay, cells (2 × 10^5^/well) were seeded into six‐well plates and grown to confluence. A vertical scratch was made using a 200 μL pipette tip, and loose cells were washed away with PBS. The remaining cells were cultured in a serum‐free medium for 24 h, and the wound area was observed and photographed under a microscope.

For the Transwell invasion assay, Matrigel was diluted (1:8) and applied to the upper chambers of the Transwell inserts. Once the gel solidified, the cells were suspended at a density of 5 × 10^5^ cells/mL, and 200 μL of the suspension was added to the upper chamber. The lower chamber was filled with 600 μL of medium containing 10% FBS. After 24 h of incubation at 37°C, the cells were fixed with formaldehyde, stained with 0.5% crystal violet for 10 min, and examined under a microscope.

### 2.4 TUNEL Staining

Cell‐loaded coverslips in culture plates were washed three times with saline and then fixed with 4% paraformaldehyde for 15 min. Mouse thyroid cancer tissue sections were deparaffinized and dehydrated.

Subsequently, 50 μL of TUNEL detection solution was applied, and samples were incubated at 37°C in the dark for 60 min. After three 10 min washes with PBS, the coverslips were mounted with an antifade medium containing DAPI and viewed under a fluorescence microscope.

### 2.5 ROS Assay

Intracellular ROS levels were measured using an ROS assay kit (S0033S, Beyotime, Shanghai, China). The cells were incubated with 10 mM DCFH‐DA for 30 min at 37°C in the absence of light and washed three times. Subsequently, intracellular ROS production was measured with flow cytometry and fluorescence microscopy (Leica, MHG, Germany).

### 2.6 Mitochondrial Membrane Potential Detection

To assess mitochondrial membrane potential, the cells were incubated with a JC‐1 fluorescent probe for 20 min at 37°C. The supernatant was then removed, and the cells were washed twice with JC‐1 buffer solution. Then, 2 mL of a cell culture medium, which included serum and phenol red, was added to each sample. The samples were then analyzed using a fluorescence microscope.

### 2.7 Animal Experiment

Female BALB/c nu/nu mice (3–5 weeks old, 14–19 g) were obtained from Beijing Vital River Laboratory Animal Technology Co. Ltd. The mice were housed in a clean and sterile plastic cage with 25°C + 2°C temperature and 55% humidity. The animals had free access to water and food for a week to adapt to the environment.

Subcutaneous injections of TPC‐1 cells (4 × 10^6^) were administered to the mice. The xenograft mice were randomly divided into four groups consisting of 10 mice each: model control (saline), taxol (treated with 5 mg/kg taxol via intraperitoneal injection), IDET (treated with 10 mg/kg IDET via intraperitoneal injection), and IDET+taxol groups (treated with 10 mg/kg IDET and 5 mg/kg taxol via intraperitoneal injection). In addition, experimental groups (treated with 50, 100, and 200 mg/kg QUE formulated in 0.5% CMC‐Na via intragastric administration) and a positive group (treated with 50 mg/kg 5‐FU formulated in saline) were established. All treatments were administered once daily for 21 consecutive days.

### 2.8 Histological Analysis

For tumor growth measurement, tumor length and width were measured every 3 days with a caliper at 5 days post‐inoculation. Tumor volume was calculated using the following formula: tumor volume (mm^3^) = [length (mm) × width (mm) × width (mm)]/2. A tumor growth curve was then plotted.

At the end of the treatment period, the mice were euthanized through cervical dislocation, the dorsal skin was incised, and the tumors were carefully excised and weighed for the calculation of the tumor inhibition rate (%) using the following formula: inhibition rate = [(tumor weight in the control group—tumor weight in the treatment group)/tumor weight in the control group] × 100%.

Blood samples were collected from the retro‐orbital sinus for analysis of liver and kidney function, and complete blood counts were obtained using an automated biochemical analyzer.

Tumor tissues and major organs were fixed in 4% paraformaldehyde, dehydrated, embedded in paraffin, sectioned, and stained with hematoxylin–eosin (HE) solution. Histological changes in the tumor and organ tissues were observed under a microscope.

Immunofluorescence staining was conducted using DAPI and mouse anti‐PCNA antibodies with a goat anti‐mouse secondary antibody. The percentage of PCNA‐positive cells was analyzed using AxioVision software. Immunohistochemical analysis of Ki67 protein expression was performed. Slides were placed in a diluted sodium citrate antigen‐retrieval solution, heated to boiling, and then boiled gently for 20 min. After cooling to room temperature, the slides were treated with 3% hydrogen peroxide to block endogenous peroxidase activity. The primary antibody was incubated overnight at 4°C in a humidified chamber, and then the slides were incubated with HRP‐conjugated secondary antibodies for 50 min at room temperature, stained with DAB staining, counterstained with hematoxylin, differentiated, and subjected to bluing treatment. The slides were then dehydrated, mounted with neutral balsam, and observed under a microscope.

### 2.9 Real‐Time PCR (qRT‐PCR)

Total RNA was extracted with Trizol reagent, and reverse‐transcribed with an InRcute IcRNA cDNA first strand synthesis kit from Tiangen. All procedures were carried out on ice. The thawed template RNA and reagents were promptly placed on ice. After mixing each solution thoroughly, a genomic DNA‐removal mix was prepared, incubated at 42°C for 3 min, and immediately placed on ice. The reverse transcription reaction mix was then prepared and incubated at 42°C for 15 min and at 95°C for 3 min. After incubation, the cDNA was stored on ice. PCR reactions were prepared using Thermo Fisher's PowerUp SYBR Green Master Mix, and the primers were synthesized by Zhejiang Shanya Biotechnology Co. Ltd. The sequences are listed in the Table 1. Thermal cycling conditions involved predenaturation, denaturation, annealing, and melting for a total of 41 cycles. Each gene was tested in three replicate wells, and three independent experiments were conducted. GAPDH was used as an internal control, and relative gene expression levels were calculated using the 2^−ΔΔCt^ method.

**TABLE 1:** Primer sequences.

Gene	Sequence
*GAPDH*	F: 5′‐ACAACTTTGGTATCGTGGAAGG‐3′ R: 5′‐GCCATCACGCCACAGTTTC‐3′
*KEAP1*	F: 5′‐CTGGAGGATCATACCAAGCAGG‐3′ R: 5′‐GGATACCCTCAATGGACACCAC‐3′
*NFE2L2*	F: 5′‐TCAGCGACGGAAAGAGTATGA‐3′ R: 5′‐CCACTGGTTTCTGACTGGATGT‐3′
*NQOI*	F: 5′‐GAAGAGCACTGATCGTACTGGC‐3′ R: 5′‐GGATACTGAAAGTTCGCAGGG‐3′
*HMOXI*	F: 5′‐CTTTGAGGAGTTGCAGGAGC‐3′ R: 5′‐TGTAAGGACCCATCGGAGAA‐3′
*GCLM*	F: 5′‐CATTTACAGCCTTACTGGGAGG‐3′ R: 5′‐ATGCAGTCAAATCTGGTGGCA‐3′
*FTH1*	F: 5′‐TCCTACGTTTACCTGTCCATGT‐3′ R: 5′‐GTTTGTGCAGTTCCAGTAGTGA‐3′

### 2.10 Western Blot Analysis

Total proteins from cells were extracted using a RIPA lysis buffer, and nuclear proteins were extracted with a nuclear protein extraction kit. The protein concentrations were determined using an enhanced BCA protein assay kit, and the samples were prepared for electrophoresis. Then, 25 μg of protein per sample was separated with 10% SDS‐PAGE and then transferred onto a PVDF membrane. The membrane was incubated in 5% BSA for 2 h and incubated overnight at 4°C with primary antibodies: Bcl‐2 (1:1000), Bax (1:1000), caspase‐3 (1:1000), Keap1 (1:1000), Nrf2 (1:1000), p62 (1:1000), Histone H3 (1:1000), or β‐Actin (1:5000). After washing with TBST, the membrane was incubated at room temperature in the dark for 1 h with an HRP‐conjugated anti‐rabbit IgG secondary antibody (1:4000). After incubation with a secondary antibody, the membrane was washed again with TBST, treated with a SuperSignal chemiluminescent substrate, and visualized using a gel imaging system.

### 2.11 Statistical Analysis

All data were statistically analyzed using GraphPad Prism 9.0 and SPSS 21.0 software. The results were presented as mean ± standard deviation. Differences between groups were assessed using one‐way analysis of variance (one‐way ANOVA). *p* < 0.05 was considered statistically significant.

## 3 Results

### 3.1 IDET Exerts Anti‐Thyroid Cancer Effects and Enhances Taxol Chemosensitivity

**FIGURE 1:**
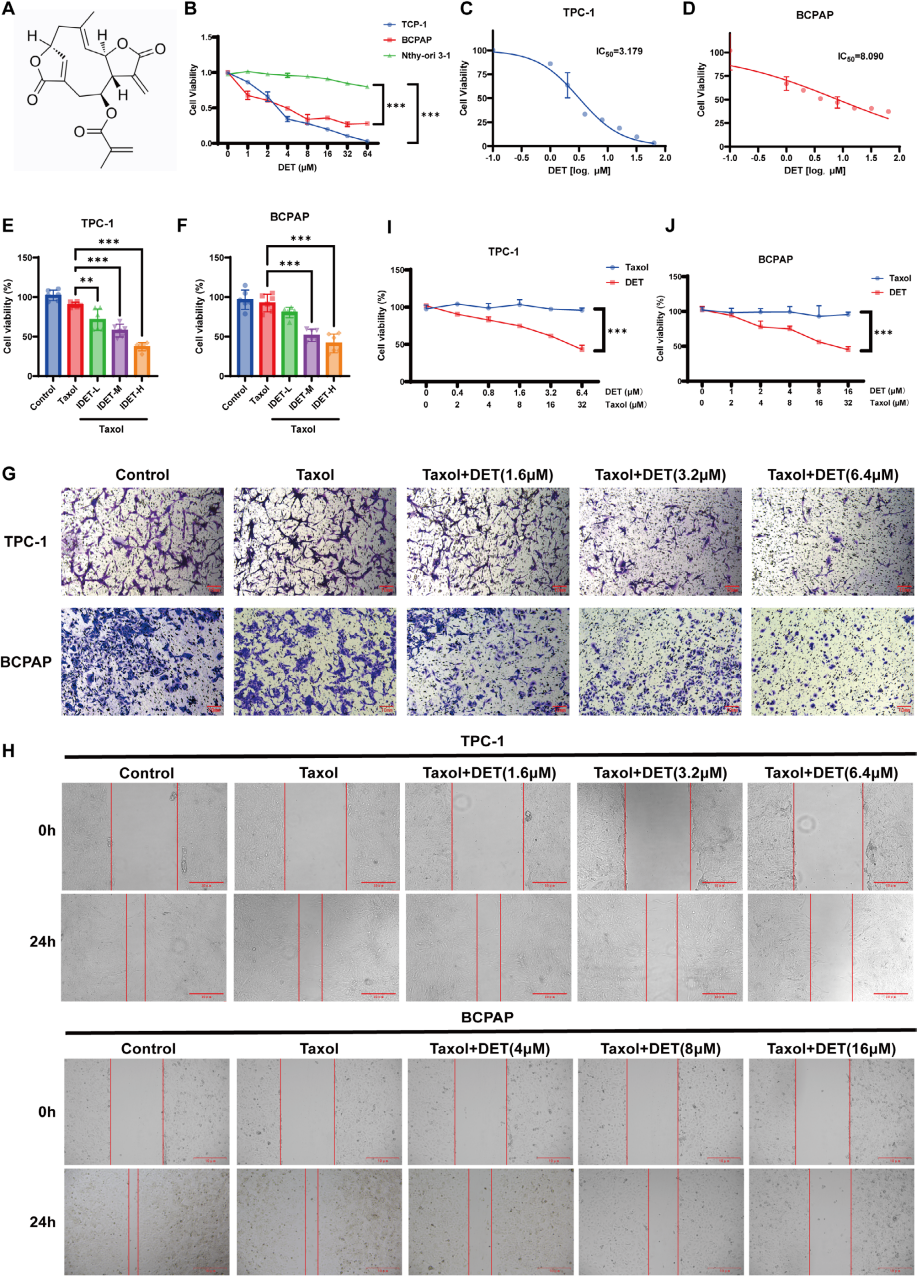
IDET exhibits anti‐thyroid cancer effects and enhances the chemotherapy sensitivity to taxol. (A) Chemical structure of IDET. (B) Effect of different concentrations of IDET on the proliferation of thyroid cancer cell lines TCP‐1 and BCPAP, as well as normal human thyroid cells Nthy‐ori 3–1. (C, D) IC_50_ values for IDET in TCP‐1 and BCPAP cells. (E, F) Assessment of cell viability in TCP‐1 and BCPAP cells treated with different IDET concentrations and/or 4 nM taxol over 24 h using CCK‐8 assays. (G, H) Transwell invasion and scratch assays conducted on TCP‐1 and BCPAP cells treated with different concentrations of IDET and/or 4 nM taxol. (I, J) The synergistic effects of IDET combined with taxol were evaluated using CCK‐8 assays in TCP‐1 and BCPAP cells. All values are reported as mean ± SD, *n* = 6, **p < 0.01, ***p < 0.001.

We first evaluated the cytotoxicity of IDET by treating the thyroid cancer cell lines TCP‐1 and BCPAP and the normal human thyroid cell Nthy‐ori 3‐1 with IDET at concentrations ranging from 1 to 64 μM. A CCK‐8 assay was used to measure cell viability. IDET displayed dose‐dependent cytotoxicity against TCP‐1 and BCPAP cells, while exerting minimal inhibitory effects on Nthy‐ori 3‐1 cells, demonstrating high selectivity for killing thyroid cancer cells (Figure 1A–B). The half‐maximal inhibitory concentrations of IDET for TCP‐1 and BCPAP were 3.179 and 8.09 μM, respectively (Figure 1C–D). The cells were then pretreated with a low dose of taxol (4 nM), and TCP‐1 cells were treated with IDET at low (1.6 μM), medium (3.2 μM), and high (6.4 μM) doses, whereas BCPAP cells were treated with low (4 μM), medium (8 μM), and high (16 μM) doses for 24 h each. The effect of IDET was further evaluated using cell proliferation, transwell invasion, and scratch assays. The results demonstrated that IDET significantly suppressed the proliferation, migration, and invasion of the cancer cells (Figure 1E–H). To establish the chemosensitizing effects of IDET, we developed taxol‐resistant thyroid cancer cell lines TCP‐1/taxol and BCPAP/taxol. Taxol alone was nearly ineffective against these cell lines, whereas IDET inhibited the proliferation of the resistant cells (Figure 1I–J). These findings confirmed the chemosensitizing role of IDET in enhancing the effect of taxol against resistant thyroid cancer cells.

### 3.2 IDET Enhances Taxol Sensitivity by Increasing Intracellular ROS Levels

**FIGURE 2:**
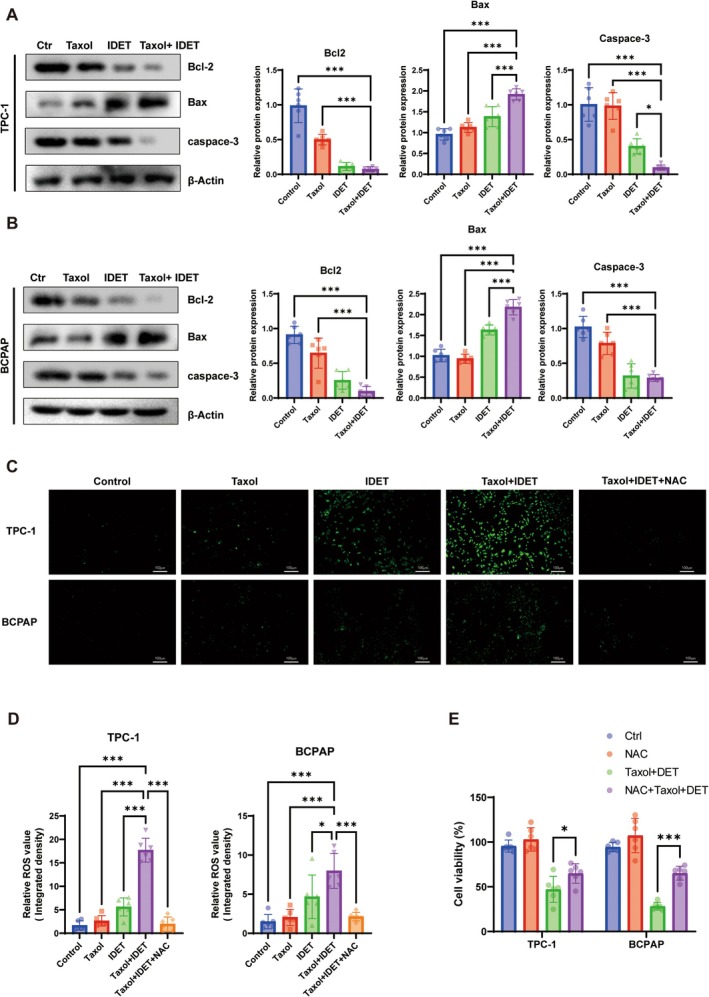
IDET enhances the sensitivity of cancer cells to taxol by increasing intracellular ROS levels. (A, B) Effect of IDET and/or 4 nM taxol treatment on the levels of apoptosis‐related proteins in TCP‐1 and BCPAP cells. (C, D) Assessment of intracellular ROS levels in TCP‐1 and BCPAP cells treated with IDET and/or 4 nM taxol using DCFH‐DA probes. (E) Assessment of cell viability in TCP‐1 and BCPAP cells treated with antioxidant NAC and/or IDET + Taxol over 24 h using CCK‐8 assays. All values are reported as mean ± SD, *n* = 6, *p < 0.05, **p < 0.01.

To further investigate the mechanism by which IDET enhances taxol chemosensitivity, we analyzed whether IDET can synergistically enhance taxol‐induced cell apoptosis. Western blot results showed that compared with treatment with IDET or taxol alone, treatment with both agents considerably increased the pro‐apoptotic protein Bax, while reducing the levels of the anti‐apoptotic proteins Bcl‐2 and caspase‐3 (Figure 2A–B). These results indicated that a combination of IDET and taxol effectively induced apoptosis in the thyroid cancer cell lines TCP‐1 and BCPAP. Excessive intracellular ROS can induce oxidative stress. This, in turn, initiates apoptotic signaling cascades. Thus, to investigate whether IDET enhances taxol sensitivity through ROS, we measured the intracellular ROS levels pre‐ and post‐IDET treatment. Our results showed that compared with either agent alone, a combination of IDET and taxol further increased ROS levels in the cells (Figure 2C–D). To confirm the involvement of ROS in this process, we used NAC, which is an ROS‐specific inhibitor, to determine its effect on IDET in thyroid cancer cells. CCK‐8 assays indicated that in the presence of taxol, cell viability was significantly higher in the NAC + IDET+taxol group than in the IDET+taxol group (Figure 2E) and intracellular ROS levels decreased (Figure 2C–D). These findings revealed that NAC can significantly reduce IDET‐induced oxidative stress, confirming that IDET‐induced increase in ROS levels plays a pivotal role in enhancing the chemosensitivity of thyroid cancer cells to taxol.

### 3.3 IDET Modulates ROS Levels in Thyroid Cancer Cells via the p62‐Keap1‐Nrf2 Pathway

**FIGURE 3:**
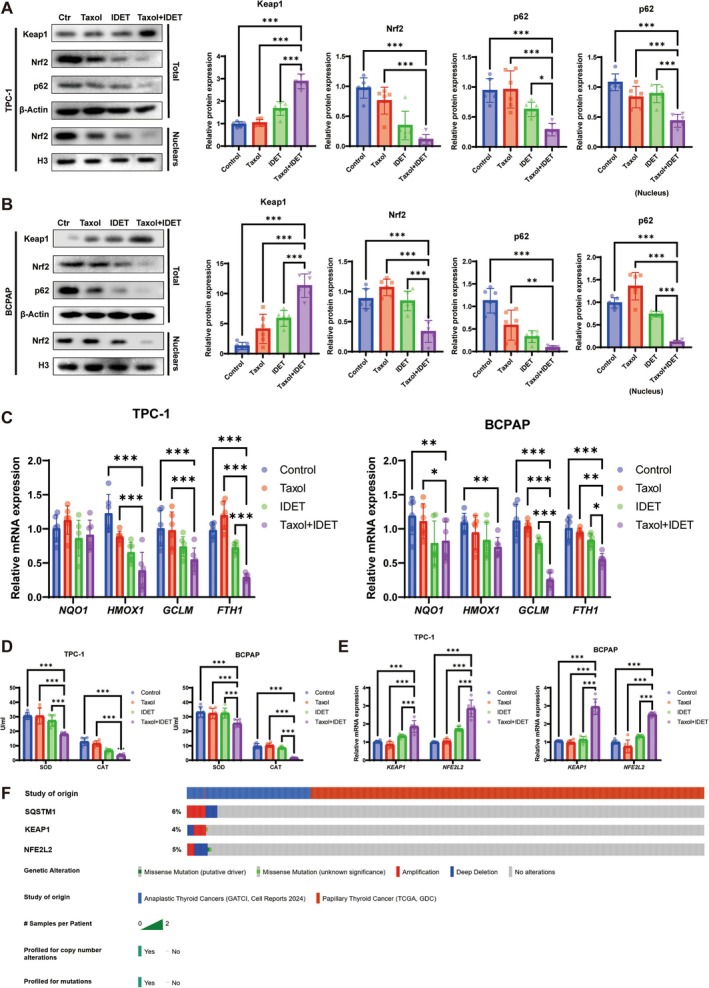
IDET regulates intracellular ROS levels in thyroid cancer cells through the p62‐Keap1‐Nrf2 signaling pathway. (A, B) Western blot assessment of the effects of IDET and/or 4 nM taxol on the p62‐Keap1‐Nrf2 signaling pathway in TCP‐1 and BCPAP cells. (C) qRT‐PCR assessment of the effect of IDET and/or 4 nM taxol on the mRNA expression of antioxidant‐related genes *NQO1*, *HMOX1*, *GCLM*, and *FTH1* in TCP‐1 and BCPAP cells. (D) ELISA assessment of the effect of IDET combined with taxol on the activities of antioxidant enzymes SOD and CAT in the supernatants of TCP‐1 and BCPAP cells. (E) The influence of IDET combined with taxol on the mRNA expression of *KEAP1* and *NFE2L2* in TCP‐1 and BCPAP cells. (F) cBioPortal analysis of mutations in *SOSTM1*, *KEAP1*, and *NFE2L2* in the tumor cells of thyroid cancer patients. All values are reported as mean ± SD, *n* = 6, **p < 0.01, ***p < 0.001.

The regulation of intracellular ROS levels is closely associated with the nuclear transcription factor Nrf2, which controls the expression of various antioxidant enzymes. The p62‐Keap1‐Nrf2 signaling pathway is essential for maintaining redox balance and providing protection against oxidative stress in the body, and its activation can promote tumor growth and resistance through Nrf2‐driven metabolic pathways. We hypothesized that IDET exerts its anti‐thyroid cancer effects and enhances taxol sensitivity by modulating the p62‐Keap1‐Nrf2 pathway, increasing intracellular ROS levels. Western blot results in TCP‐1 cells indicated that a combination of IDET and taxol more considerably downregulated p62 protein expression while upregulating Keap1 protein levels than treatment with either agent alone (Figure 3A). Additionally, Nrf2 protein levels in the cytoplasm and nucleus were significantly reduced in the IDET–taxol combination group compared with the control group (Figure 3A). Similar findings were observed in BCPAP cells, where IDET combined with taxol reduced p62 and Nrf2 protein levels and considerably elevated Keap1 protein levels (Figure 3B).

Moreover, we analyzed the effect of IDET on the expression of Nrf2 downstream target genes through qRT‐PCR. In TCP‐1 cells, IDET combined with taxol did not significantly change *NQO1* mRNA expression but led to significant reductions in *HMOX1*, *GCLM*, and *FTH1* RNA expression levels (Figure 3C). In BCPAP cells, IDET combined with taxol had no significant effect on *NQO1* and *GCLM* mRNA levels but significantly decreased *HMOX1* and *FTH1* mRNA expression levels (Figure 3C). ELISA analysis of the cell supernatants from TCP‐1 and BCPAP cells revealed that SOD and CAT activity diminished in the IDET–taxol combination group compared with IDET or taxol alone (Figure 3D). Moreover, qRT‐PCR analysis showed that IDET combined with taxol considerably increased *KEAP1* and *NFE2L2* mRNA expression levels in TCP‐1 and BCPAP cells (Figure 3E).

We proceeded to analyze mutations in the *SOSTM1*, *KEAP1*, and *NFE2L2* genes in patients with thyroid cancer. cBioPortal analysis indicated that mutations in the genes encoding p62, Keap1, and Nrf2 were present in these patients, and 15% of patients with thyroid cancer exhibited mutations in *SOSTM1*, *KEAP1*, or *NFE2L2*. These mutations were primarily characterized by abnormal amplification and deep deletions in copy number (Figure 3F). This finding suggested that mutations in the genes encoding the p62‐Keap1‐Nrf2 signaling pathway are closely associated with the progression of thyroid cancer.

### 3.4 Nrf2 Activation Reverses IDET‐Induced Enhancement in Taxol Sensitivity

**FIGURE 4:**
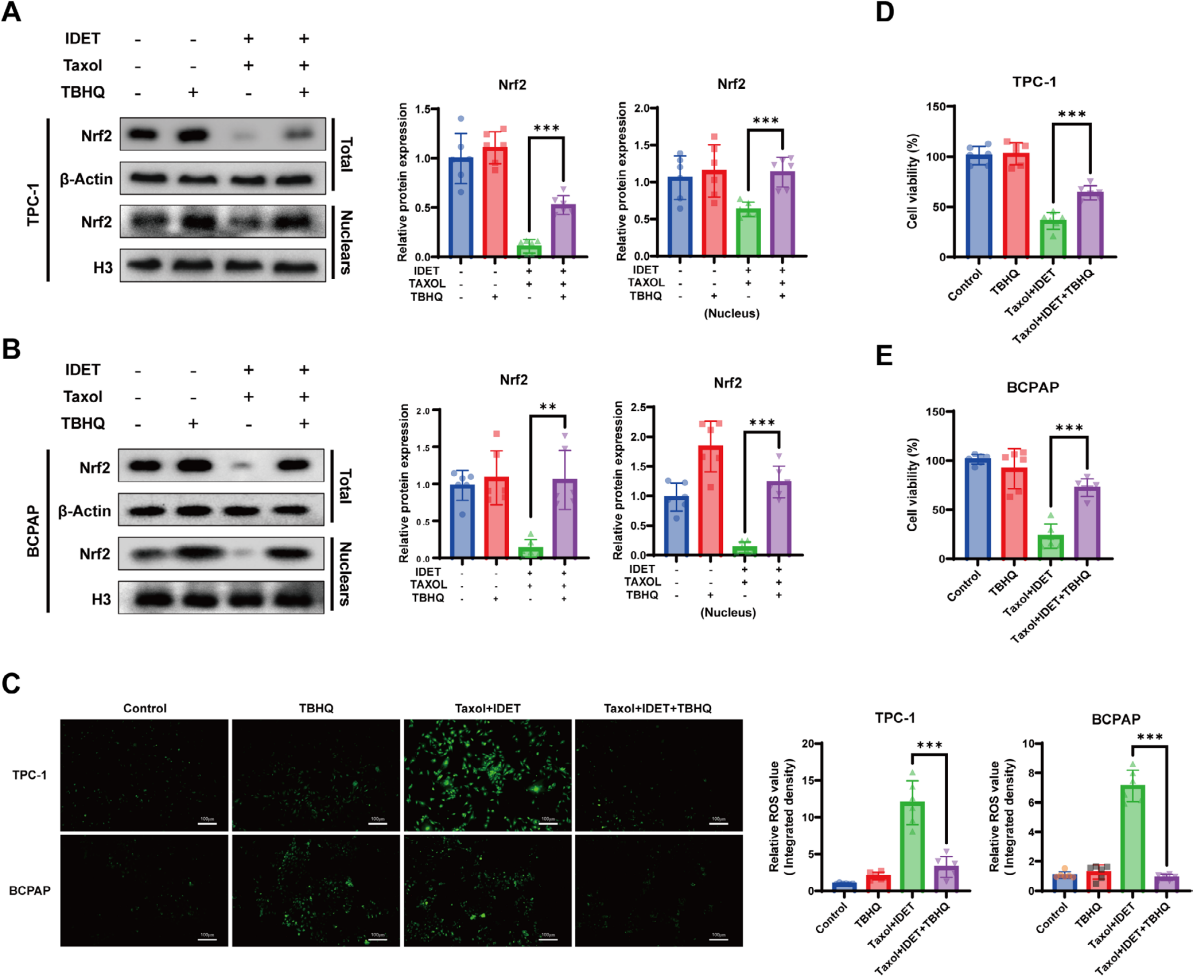
Activation of Nrf2 can block the sensitizing effect of IDET on taxol. (A, B) Western blot analysis assesses the effect of the Nrf2‐specific activator TBHQ on the expression of Nrf2 protein suppressed by IDET in combination with taxol. (C) Assessment of ROS levels in thyroid cancer cells after TBHQ treatment using the ROS probe DCFH‐DA. (D, E) CCK‐8 assays evaluate thyroid cancer cell proliferation after TBHQ intervention. All values are reported as mean ± SD, *n* = 6, **p < 0.01, ***p < 0.001.

To further explore the potential mechanism by which IDET suppresses Nrf2 expression, we employed TBHQ, which is a specific Nrf2 activator. Western blot results showed that Nrf2 protein levels were considerably higher in the TBHQ+IDET+taxol group than in the IDET+taxol group in TCP‐1 and BCPAP cells, suggesting that TBHQ can partially restore Nrf2 expression that was suppressed by IDET (Figure 4A–B). ROS analysis revealed that intracellular ROS levels were significantly lower in the TBHQ+IDET+taxol group than in the IDET+taxol group, indicating that Nrf2 activation can counteract IDET‐induced increases in ROS levels (Figure 4C). Furthermore, the CCK‐8 assay demonstrated that cell proliferation was markedly enhanced in the TBHQ+IDET+taxol group compared with the IDET+taxol group (Figure 4D–E). These results demonstrated that the IDET‐mediated activation of the p62‐Keap1‐Nrf2 signaling pathway increases ROS levels in thyroid cancer cells and results in the oxidative stress–induced inhibition of cell proliferation and enhanced sensitivity to taxol.

### 3.5 IDET Enhances Chemosensitivity to Taxol in Thyroid Cancer Mice

**FIGURE 5:**
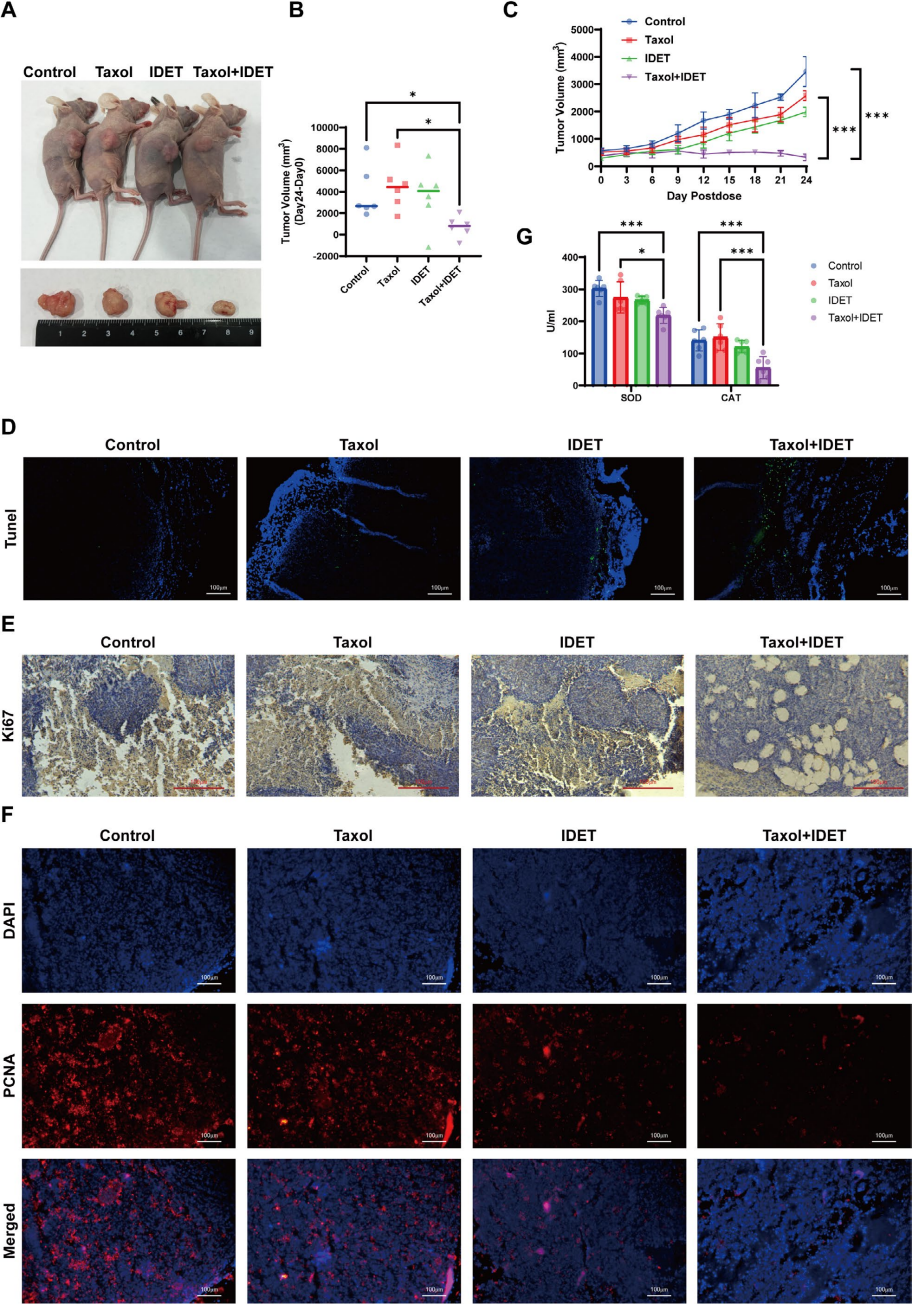
IDET enhances the sensitivity of thyroid cancer xenograft mice to taxol chemotherapy. (A) Representative images of xenograft nude mice. (B) Weights of tumors in nude mice. (C) Growth curves of tumors in nude mice. (D) TUNEL/DAPI dual staining to evaluate apoptosis levels in tumors from nude mice. (E) Immunohistochemical examination of Ki67 protein expression in the xenograft tumor samples. (F) Immunofluorescence analysis of PCNA protein expression in xenograft tumor tissues. (G) ELISA assessment of SOD and CAT activities in the serum of xenograft nude mice. All values are reported as mean ± SD, *n* = 6, *p < 0.05, ***p < 0.001.

On the basis of our in vitro results, we aimed to verify the synergistic effects of IDET and taxol in vivo. A thyroid papillary carcinoma xenograft model was established by subcutaneously injecting TCP‐1 cells into BALB/c‐nu/nu mice. Treatment with 10 mg/kg taxol resulted in a modest antitumor effect. However, a combination of IDET and taxol inhibited tumor growth (Figure 5A–C). TUNEL assays indicated that tumor tissues treated with a combination of IDET and taxol (Figure 5D) had higher rates of apoptosis than those treated with either agent alone. Immunohistochemical analysis further confirmed that Ki67 expression was markedly reduced when IDET and taxol were administered together (Figure 5E). Additionally, proliferating cell nuclear antigen (PCNA) fluorescence staining indicated a decrease in PCNA‐positive cells at the tumor site treated with a combination of IDET and taxol (Figure 5F). Given that our in vitro studies suggested that oxidative stress–induced apoptosis plays a key role in the chemosensitization effect of IDET, the in vivo experiments indicated that IDET triggers apoptosis in thyroid cancer cells within xenograft tumors. To further confirm the link between apoptosis and oxidative stress, we employed an ELISA to measure SOD and CAT activity in the sera of tumor‐bearing mice. The results showed a significant reduction in SOD and CAT activity in the serum of mice treated with the IDET–taxol combination (Figure 5G). These findings suggested that IDET, in combination with taxol, enhances oxidative stress by decreasing antioxidant enzyme activity, thereby inducing apoptosis and necrosis in xenograft tissues and ultimately inhibiting tumor growth, in line with our in vitro findings.

### 3.6 IDET Exhibits no Significant Toxicity in Thyroid Cancer Mice

**FIGURE 6:**
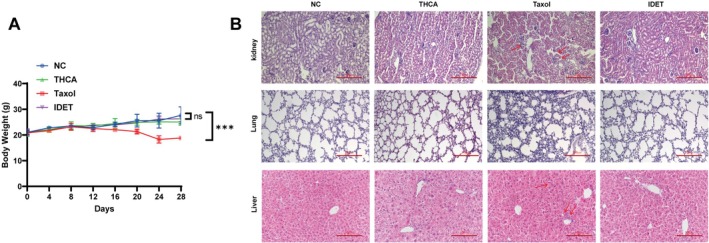
IDET exhibits no significant toxicity in thyroid cancer xenograft mice. (A) Weight change curves of xenograft nude mice. (B) Histopathological images of HE staining for the kidneys, lungs, and liver of xenograft nude mice. All values are reported as mean ± SD.

We subsequently examined the potential toxic effects of IDET in BALB/c‐nu/nu mice. During the treatment period, no abnormalities in animal behavior were observed, including activity levels, eating and drinking habits, excretion, and overall demeanor. The body weights of the mice in the IDET‐treated group did not differ significantly from those in the NC group, whereas the body weight of the mice in the taxol group compared with the NC group was significantly reduced at day 28 (Figure 6A). Peripheral blood samples were analyzed using hematological and biochemical analyzers. The results revealed no significant differences in the indicators of bone marrow function (reticulocytes, platelets, and hemoglobin), liver function (alanine aminotransferase, aspartate aminotransferase, and alkaline phosphatase), or kidney function (creatinine and blood urea nitrogen) before and after treatment (Table 2). These findings suggested that IDET does not cause significant damage to mice, whereas taxol was associated with some negative effects. Additionally, HE staining was performed on major organs (lungs, liver, and kidneys) from all groups, and their pathological changes were analyzed. The results showed no significant histopathological abnormalities in the heart, liver, or kidneys of the model group compared with the NC group, suggesting that the model mice did not induce significant toxicity, such as inflammatory cell infiltration or tissue edema, in these organs. Similarly, the IDET group exhibited no significant histopathological abnormalities in the heart, liver, or kidneys, compared with the model group, suggesting that the IDET treatment regimen used in this study did not produce considerable toxicity in the major organs of the tumor‐bearing mice. However, pathological changes, such as inflammatory cell infiltration, were observed in the kidneys of the taxol‐treated group, indicating that taxol had some level of renal toxicity in the model mice (Figure 6B).

**TABLE 2:** Analysis of blood routine parameters in xenograft nude mice.

	NC	THCA	Taxol	IDET
Hematology				
RET (10^3^/μL)	389.15 ± 43.22	502.02 ± 20.39	486.64 ± 32.85	469.12 ± 56.26
PLT‐O (10^3^/μL)	1068.16 ± 198.49	1409.26 ± 225.16	1363.64 ± 309.64	1206.34 ± 239.53
HGB (g/dL)	13.19 ± 0.60	14.02 ± 0.86	14.86 ± 0.43	15.52 ± 1.02
Hepatic funciton				
ALT (U/L)	71.50 ± 18.39	118.92 ± 86.37	129.26 ± 76.55	115.26 ± 59.24
AST (U/L)	139.27 ± 19.51	168.37 ± 45.28	197.47 ± 56.64	151.26 ± 29.06
ALP (U/L)	78.94 ± 9.67	73.00 ± 12.61	75.15 ± 13.24	64.59 ± 15.07
Renal function				
CREA (mg/dL)	0.16 ± 0.05	0.06 ± 0.01	0.04 ± 0.00	0.12 ± 0.04
BUN (mg/dL)	26.58 ± 1.36	20.14 ± 3.59	18.46 ± 5.41	27.84 ± 2.16

## 4 Discussion

Current therapeutic strategies for thyroid cancer, including surgery, radiation therapy, and chemotherapy, have certain drawbacks. Surgery, although capable of excising tumors, often leads to trauma and dependency on thyroid hormones. Radiation therapy and radioactive iodine treatment can inflict considerable damage to normal tissues, and their effectiveness is particularly limited to patients with metastatic thyroid cancer. Targeted therapies, such as sorafenib and lenvatinib, are effective but often lead to drug resistance and tumor recurrence after prolonged use (Lamartina et al. 2018). Additionally, the efficacy of single‐agent chemotherapy is generally limited, thereby prompting the use of combination therapies in preclinical and clinical studies to enhance treatment outcomes. The most used combinations are composed of taxanes (taxol and docetaxel) and carboplatin or doxorubicin (Haddad et al. 2018; Saini et al. 2018). However, combination therapies often cause severe adverse effects, such as nausea, vomiting, bone marrow suppression, and cardiotoxicity, and can induce drug resistance, complicating treatment completion (Xu et al. 2018). Therefore, exploring novel anti‐thyroid cancer agents that are effective against first‐line chemotherapy‐sensitive and resistant thyroid cancer and have minimal side effects is imperative.

Stress‐related mechanisms play crucial roles in protecting cells from damage caused by toxic agents. These stress‐related signals, such as oxidative stress, autophagy induction, and glycolysis activation, are closely linked to the development of acquired drug resistance (Pan et al. 2016). In addition, certain low‐toxicity phytochemicals have shown promising results in enhancing chemotherapy sensitivity through stress response modulation. For instance, resveratrol improves chemosensitivity in breast cancer by reversing macrophage polarization, lowering IL‐6 levels, and inhibiting STAT3 activation (Cheuk et al. 2022). Quercetin reduces tumor invasiveness and cisplatin resistance in nasopharyngeal carcinoma by regulating the Yes‐associated protein/Hippo signaling pathway (Li and Li 2023). Natural stress‐regulating molecules are promising agents for enhancing chemosensitivity while maintaining safety. In our study, we discovered that IDET enhances chemotherapy sensitivity in thyroid cancer by inducing stress overload.

IDET demonstrates a high level of antitumor activity across various types of cancer, including breast cancer (Mehmood and Muanprasat 2022), lung cancer (Beeran et al. 2015), nasopharyngeal carcinoma (Yan et al. 2013), colorectal cancer (Chan et al. 2016, 2015), and liver cancer (Mehmood et al. 2017). In this study, we confirmed the safety and antitumor efficacy of IDET through in vitro and in vivo experiments using cultured thyroid cancer cells and a thyroid cancer xenograft mouse model. IDET inhibited the proliferation, migration, and invasion of two thyroid cancer cell lines in a dose‐dependent manner without causing toxicity to normal thyroid cells. In the thyroid cancer xenograft mouse model, IDET treatment had no substantial effect on body weight or major organs, and no notable toxicity was observed in liver, kidney, or bone marrow function. This finding confirmed that IDET is safe for normal thyroid cells and exhibits selective cytotoxicity against thyroid cancer cells. Furthermore, we demonstrated that combining IDET with taxol enhances chemotherapy efficacy while mitigating side effects. In the taxol‐resistant thyroid cancer cell lines TCP‐1/taxol and BCPAP/taxol, IDET inhibited proliferation and induced apoptosis. In the thyroid cancer xenograft mouse model, a combination of IDET and taxol displayed excellent antitumor effects. These findings suggest that IDET enhances the chemosensitivity of thyroid cancer to taxol, thereby serving as an additional treatment option for the clinical management of thyroid cancer.

IDET modulates various molecular targets, including NF‐κB, PI3K/AKT/mTOR, Wnt/β‐catenin, STAT3, and MAPK, thereby influencing processes such as apoptosis, autophagy, cell cycle regulation, and cell survival, which in turn affect tumor progression (Mehmood and Muanprasat 2022). Cancer cells are in an elevated metabolic state and thus generally exhibit higher oxidative stress levels than normal cells. This feature ensures the survival and proliferation of cancer cells. Excessive intracellular ROS accumulation can lead to apoptosis and cell cycle arrest, thereby inhibiting cancer cell proliferation (Havrdová et al. 2022). Given the sensitivity of tumor cells to ROS, at least four chemotherapeutic agents, namely, cisplatin, doxorubicin, arsenic trioxide, and bleomycin, kill cancer cells by enhancing ROS production (Gao et al. 2022). To explore whether IDET can regulate redox homeostasis and thereby influence the drug sensitivity of thyroid cancer cells, we examined intracellular ROS levels and mitochondrial membrane potential after IDET treatment. The results demonstrated that IDET, in combination with taxol, considerably increased ROS levels in thyroid cancer cells and induced mitochondrial depolarization (indicated by a significantly decreased mitochondrial membrane potential). Furthermore, the use of NAC (a ROS scavenger) in an intervention experiment confirmed these findings. The results showed that 2 h of NAC pretreatment partially reversed the effects of IDET on ROS levels, mitochondrial membrane potential, and apoptosis in the TCP‐1 and BCPAP cells, suggesting that the pharmacological effects of IDET in these thyroid cancer cells are mediated by ROS. Mitochondria in cancer cells produce high levels of ROS, which can disrupt mitochondrial oxidative phosphorylation and lead to mitochondrial dysfunction and apoptosis. Excessive intracellular ROS can impair mitochondrial oxidative phosphorylation in cancer cells, thereby causing apoptosis. Therefore, we propose that IDET enhances the chemosensitivity of TCP‐1 and BCPAP cells by inducing ROS accumulation that promotes mitochondrial damage and apoptosis.

Oxidative stress activation indicates an overabundance of free radicals within the body. Redox homeostasis is maintained by endogenous antioxidant enzymes that eliminate ROS. The nuclear transcription factor Nrf2 promotes the expression of antioxidant enzymes and repairs proteins damaged by oxidative stress by targeting various antioxidant genes. The protein p62 competes with Keap1, preventing the degradation of Nrf2, which in turn enhances the transcription of the *SQSTM1* gene and increases p62 protein expression. These processes form a positive feedback loop involving the p62‐Keap1‐Nrf2 pathway, which maintains cellular antioxidant defenses (Gureev et al. 2020). Hence, we propose that the p62‐Keap1‐Nrf2 signaling pathway is a potential target on which IDET exerts its anti‐thyroid cancer effects. The Western blot results revealed that IDET considerably decreased p62 protein expression in thyroid cancer cells while increasing Keap1 protein expression, and these effects were accompanied by low Nrf2 protein levels in the cytoplasm and nucleus. Currently, many small molecules derived from natural plants exert antitumor effects by inhibiting Nrf2. For example, quercetin inhibits the proliferation of thyroid and lung cancer cells by suppressing NFE2L2 mRNA expression and limiting Nrf2 nuclear translocation (Mostafavi‐Pour et al. 2017). Similarly, apigenin induces apoptosis in ovarian cancer cells by inhibiting Nrf2 protein expression and promoting ROS production (Zhao et al. 2021). In this study, we demonstrated that IDET‐induced increase in ROS levels in thyroid cancer cells is closely associated with the downregulation of Nrf2 protein expression.

Nrf2 translocates into the nucleus and eliminates ROS by mediating the expression of phase II detoxifying and antioxidant enzymes via the ARE. NQO1 and HO‐1 are phase II enzymes that effectively provide protection against oxidative substances through different mechanisms (Zhang et al. 2021). Moreover, GCLM in the Nrf2 pathway enhances the biosynthesis of GSH, thereby improving tumor cell resistance to ROS, which can affect tumor invasion and resistance to chemotherapy (Lin et al. 2023). Moreover, Nrf2 activates the transcription of *FTH1*, serving as an antioxidant to protect cells from oxidative damage (Muhoberac and Vidal 2019). Our results confirmed that a combination of IDET and taxol reduces the mRNA expression of *NQO1*, *HO‐1*, *GCLM*, and *FTH1* in thyroid cancer cells. Furthermore, SOD and CAT, which are downstream antioxidant enzymes in the Nrf2 signaling pathway, can scavenge ROS and protect cells from oxidative stress damage. The ELISA assays revealed that IDET markedly reduced SOD and CAT activity in the supernatant of thyroid cancer cells, indicating its inhibitory effect on Nrf2 function. In conclusion, IDET restricts Nrf2 nuclear translocation, suppresses its downstream antioxidant functions, including the expression of *HMOXI*, *FTH1*, *GCLM*, SOD, and CAT, and resulting in ROS accumulation within cells. Notably, qRT‐PCR analysis showed that IDET considerably upregulated the expression of *KEAP1* and *NFE2L2* mRNAs in thyroid cancer cells, where Nrf2 is encoded by *NFE2L2*. Therefore, IDET exerts its inhibitory effect on Nrf2 protein expression through post‐translational modifications rather than through transcriptional regulation, leading to a compensatory increase in *NFE2L2* mRNA expression. TBHQ, which is a specific activator of Nrf2, partially restored Nrf2 protein expression downregulated by IDET. Finally, using Nrf2‐overexpressing TCP‐1 and BCPAP cell lines, we found that the overexpression of Nrf2 reverses the IDET‐induced inhibition of Nrf2 protein expression and the associated increase in ROS levels and inhibition of cell proliferation, thereby linking the inhibitory effect of IDET on Nrf2 to oxidative stress damage. This study clarified the molecular mechanisms by which IDET increases ROS levels in thyroid cancer cells. By downregulating p62 protein expression, upregulating Keap1 protein expression, reducing Nrf2 nuclear translocation, and inhibiting the expression of Nrf2 downstream antioxidant factors, IDET ultimately increases ROS levels, inhibits thyroid cancer cell proliferation, and enhances taxol sensitivity.

## 5 Conclusion

This study provides a comprehensive examination of the antithyroid cancer efficacy of the compound IDET isolated from *Elephantopus scaber* in vitro and in vivo, laying a strong scientific foundation and theoretical basis for its potential therapeutic application to thyroid cancer treatment. In this study, treatment combining IDET and taxol downregulated p62 expression, upregulated Keap1 expression, reduced Nrf2 nuclear translocation, and subsequently inhibited the expression of Nrf2 downstream antioxidant‐related genes and enzymes. These effects led to an increase in intracellular ROS levels and mitochondrial depolarization, ultimately inducing apoptosis in thyroid cancer cells and enhancing their sensitivity to taxol. Additionally, a combination of IDET and taxol effectively inhibited the growth of transplanted tumors in tumor‐bearing nude mice, whereas IDET alone exerted no considerable effects on the major organs and blood parameters of the mice, possessing a higher safety profile than taxol. This finding corroborated the regulatory effect of IDET on the p62‐Keap1‐Nrf2 pathway. Our study demonstrated that IDET sensitizes thyroid cancer cells to chemotherapy by directly targeting the p62‐Keap1‐Nrf2 axis and activating ROS‐mediated apoptotic pathways, thereby unveiling a novel mechanism through which IDET exerts its potent antitumor effects on thyroid cancer.

## Author Contributions

**Wei Cong:** conceptualization (equal), investigation (equal), methodology (equal), project administration (equal), supervision (equal), writing – original draft (equal), writing – review and editing (equal). **Jingfu Sun:** data curation (equal), formal analysis (equal), investigation (equal), methodology (equal). **Zhanyu Hao:** investigation (equal), methodology (equal). **Maosong Gong:** investigation (equal), methodology (equal).

## Ethics Statement

This study was approved by the Animal Ethics Committee of the Second Hospital of Shandong University (Ethical Approval No. KYLL202787).

## Conflicts of Interest

The authors declare no conflicts of interest.

## Data Availability

The data that support the findings of this study are available from the corresponding author upon reasonable request.
